# Color of the Blood

**Published:** 1845-05

**Authors:** 


From Mr. Paget's learned Report on the Progress of Human Anatomy and Physiology in the years 1843--4, in the British and Foreign Medical Review, we make the following extracts:

*Color of the Blood.*—Some experiments by Scherer both confirm the opinion of Nasse, that the change from the arterial to the venous colour of the blood depends in a great measure on the form of the blood-corpuscles, and explain most of the observations of Dr. Stevens on the effects of distilled water and salts upon the blood. Their general conclusions are; 1. That when fresh-stirred and bright-red ox-blood is mixed with distilled water, it acquires a dark-red colour, and its corpuscles, by imbibing water, become spherical, and at last vanish. But, 2, That if, after the change has begun, and not gone far, a concentrated solution of a neutral salt be added, the blood-corpuscles again acquire their natural form, and the bright-red colour is restored. 3. That when oxygen is passed through blood darkened by the addition of distilled water, it is not changed in colour, and the blood corpuscles do not reappear; but that the same kind of blood, mixed with a small quantity of milk, or oil, or finely-powdered chalk, or gypsum, soon regains its bright-red colour. 4. Again, by the long-continued contact of concentrated saline solutions with blood-corpuscles they become jagged and decomposed, and the blood becomes black; and those which have been reddened by the action of salts, become black again on being expanded by the imbibition of water. 5. By adding carbonic acid to bright-red blood, its corpuscles change their biconcave for a biconvex form, and at the same time its colour changes from red to black. So that there are always changes in the shape of the blood corpuscles, coincident with the changes in the colour of the mass of blood; whenever they are dilated, as by distilled water or carbonic acid, the dark colour is produced; whenever they are contracted into the biconcave form, the bright-red colour is restored.

Mulder, also, espouses the opinion of the changes of colour in the blood being immediately due to physical rather than to chemical changes of the corpuscles, and has added many facts to those just quoted in disproof of the opinion of Liebig, that the changes are due to the alternate production or the carbonate of the protoxyde, and of the peroxyde, of iron in the blood-corpuscles, as they pass alternately through the systemic and the pulmonary capillaries. His chief facts are—1. That the elementary composition of the colouring matter is the same, whether obtained from arterial or venous blood, viz., C. 44, H. 44, N. 6, O. 6, Fe. 2. That the change from dark to bright blood is effected as completely by the agency of a neutral salt as by oxygen. 3. That if the iron were present in the blood as an oxyde (and especially as a peroxyde), it should be easily extracted by weak acids; but he has found that well prepared hæmatine may be digested in diluted hydrochloric or sulphuric acid for several days without the iron in it being in the least diminished. After being so treated he has obtained, after incineration, the regular proportion of 9-49 per-cent. of oxyde. If strong sulphuric acid be poured on dried blood, or dried pure hæmatine, and kept on it for some days, and then water be added, hydrogen is evolved, and sulphate of peroxyde of iron is found in the solution, which could not happen if the iron had been at first in the form of peroxyde. 5. The iron may thus be all extracted from the blood, or from hæmatine, (though not as some say, without affecting the colour,) and the other constituents may be obtained in a separate form. Numerous analyses of this constituent, by Van Goudover, regularly yielded the same equivalents of the elements, viz., C. 44, H. 44, N. 6, O. 6; but if the iron had been united with this in the form of Fe. 2, O. 3, and in the proportion of one equivalent to two, there should have remained only four and a half equivalents of oxygen.

Mulder concludes, therefore, that iron is present in hæmatine, as iodine is in sponge, or sulphur in cystine, or arsenic in cacodyl. His notion of the mode in which the changes of colour are effected is, that when the corpuscles of the venous blood are exposed in the lungs, oxy-proteine is formed by the oxydation of the fibrine-proteine of the liquor sanguinis, or, perhaps, by the oxydation of the outer layer of the cell membrane of the corpuscles. If formed on the liquor sanguinis, its peculiar plasticity would lead to its being deposited in a thin layer on the corpuscles. In either such case, the dark corpuscles would, after respiration, be invested by a thin layer of white and imperfectly transparent oxy-proteine, or buffy coat, through which they would look bright red, as dark blood does when contained in a vessel of milk-white glass. But, in the systemic capillaries, the oxy-proteine may be consumed in nutrition, and the darkness of the corpuscles will then again appear unveiled.

Moreover, since it appears that, in the biconcave form, the corpuscles by reflecting more light, are always bright, and in the biconvex form always dark, it may be that in the arteral blood they are not only *buffed* but also *cupped*, by the oxy-proteine, [by the plastic properties of which, morover, it is easy, on this pretty theory, to explain the ready adhesion of the corpuscles in inflammatory blood.] Diluted acids, which make bright blood dark, may do so by making the outer layer of the corpuscles transparent, as they do fibrine before dissolving it; and concentrated solutions of neutral salts may make it bright by making the same layer contract.

